# Diagnostic Pitfalls and Unique Radiological Insights in Thyroid Paraganglioma: A Case Report and Literature Review

**DOI:** 10.1155/crot/5395659

**Published:** 2025-10-25

**Authors:** Ainulakbar Mughal, Fatima Syed Amanullah, Zubia Ali, Shabbir Akhtar, Sehar Suleman

**Affiliations:** Department of Otolaryngology, Aga Khan University Hospital, Karachi, Sindh, Pakistan

**Keywords:** case report, head and neck, immunohistochemistry, paraganglioma, thyroid

## Abstract

Paragangliomas (PGLs) are extremely rare endocrine tumors that arise from the autonomic nervous system. Their rarity contributes to their frequent misdiagnosis. Ultrasound and immunohistochemical findings are heavily relied on for correct diagnosis. We present a case of thyroid PGL in a 40-year-old female patient who presented with a 1-year history of anterior neck mass and a 4-month history of hemoptysis. Ultrasound findings showed a solitary, lobulated, hypoechoic, and vascular lesion in the right thyroid lobe. She underwent total thyroidectomy and tracheal end-to-end anastomosis. Histopathology showed cells organized in distinct nests (zellballen) pattern enclosed by a delicate fibrovascular stroma. On immunohistochemical analysis, the tumor was positive for synaptophysin, CD56, GATA-3, and S100. The patient is stable postoperatively and disease-free. The role of ultrasound is vital in forming a correct diagnosis of thyroid PGL preoperatively. We review the current literature regarding diagnostic findings and treatment of thyroid PGLs with the aim of supplementing the findings of the thyroid PGLs that have been previously reported. The creation of a robust imaging and immunohistochemical profile for this entity is needed to combat the frequent misdiagnoses that occur with thyroid PGLs.

## 1. Introduction

Paragangliomas (PGLs), previously termed as chemodectomas, are nonmalignant carcinoid tumors which grow slowly and are derived from the autonomic nervous system [1]. They can be found along the migratory pathways of neural crest tissue during embryonic development, ranging from the base of the skull to the lower pelvic region, with the exception of the extremities [1] These tumors predominantly affect women and younger individuals who have a 30% genetic predisposition to developing PGLs [1, 2]. They constitute 0.012%–0.6% of all head and neck tumors with a malignancy potential of 4%–16%. The carotid body is the most common site (80%), followed by the jugular bulb, Jacobsen's tympanic plexus, and the vagal nerve. The nasal cavity, orbit, larynx, aortic arch, thyroid, and parathyroid glands are even rarer sights for PGLs [1].

Thyroid PGLs, in particular, are a rare entity arising from the inferior laryngeal paraganglia. Patients are usually asymptomatic with the nonfunctional thyroid nodule being diagnosed incidentally. In symptomatic cases, patients experience dysphagia, dyspnea, stridor, and hemoptysis [3]. Once a PGL has developed from the inferior laryngeal paraganglia, it gradually descends and settles lateral to the thyroid gland. In some cases, inferior laryngeal paraganglia may develop within the thyroid capsule, potentially giving rise to a different subtype of PGL known as intrathyroidal PGL [3]. From a histological perspective, the tumor exhibits cells organized in distinct nests (zellballen) pattern and is enclosed by a delicate fibrovascular stroma. Thyroid PGLs are a diagnostic dilemma and are commonly misdiagnosed as follicular neoplasm, medullary thyroid carcinoma (MTC), and neuroendocrine tumors [3].

In this paper, we describe a case of thyroid PGL with a literature review of the diagnosis and management of this entity.

## 2. Case Presentation

A 40-year-old lady, teacher by profession, with no prior co-morbid conditions presented to the otolaryngology clinic with a 1-year history of an anterior neck mass and a 4 months history of hemoptysis. There was no complaint of hoarseness.

On examination, a 4 × 3 cm firm swelling was palpated in the right thyroid lobe extending towards the sternocleidomastoid muscle. There was no tracheal shift or retrosternal extension.

Both vocal cords appeared normal on endoscopic laryngeal examination. Narrowing of the tracheal lumen in the subglottic region was noted, raising suspicion of local invasion by the thyroid gland.

**Figure 1:**
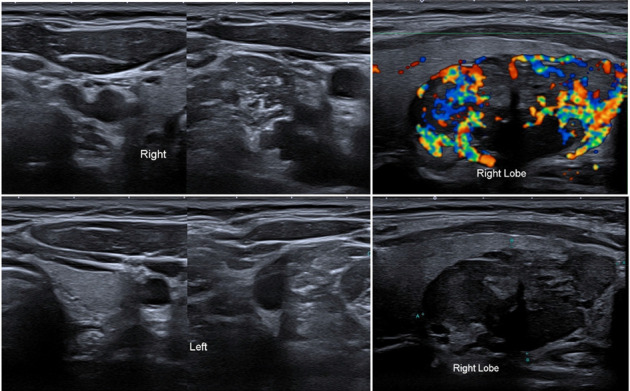
Ultrasound of the left and right lobe of the thyroid.

Ultrasound of the neck (Figure 1) showed a 4.9 × 2.2 cm solitary, lobulated, hypoechoic, and vascular lesion in the right lobe of the thyroid gland. No calcifications were seen. The rest of the right lobe appeared normal. There were no solid or cystic nodules seen in the left lobe.

There was no evidence of lymphadenopathy on either side of the neck. The right lobe of thyroid measured approximately 5.5 × 2.5 cm. The left lobe of thyroid measured approximately 3.8 × 1.0 cm. The thickness of isthmus was about 0.33 cm.

The lesion was assigned as class V by the Thyroid Imaging Reporting and Data Systems (TIRADS). Fine needle aspiration cytology (FNAC) of the nodule showed clusters and groups of follicular cells with nuclear overlapping against hemorrhagic background mixed with colloid—corresponding to class III by the Bethesda System for Reporting Thyroid Cytopathology (TBSRTC). The management plan was discussed in thyroid and parathyroid tumor board and with the patient. The plan included possible tracheal resection in case of gross involvement and the patient agreed.

Intraoperatively, a very vascular 4 × 2 cm mass involving the right thyroid lobe and extending to the trachealis muscle posteriorly was found. It had also eroded the cricoid cartilage. The tumor was excised with tracheal rings and tracheal end to end anastomosis was done. Bilateral recurrent laryngeal nerves were identified and saved. The right superior laryngeal nerve was not seen. The patient was kept in the surgical ICU for one day postoperatively. She had an uneventful postoperative period. Her voice was not hoarse and calcium levels were normal.

**Figure 2:**
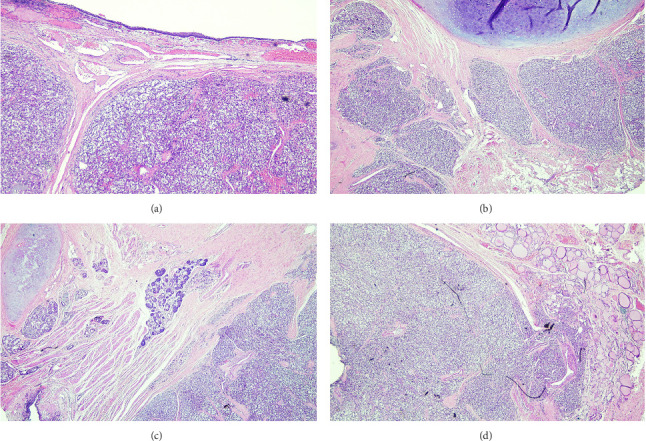
(H&E 4x). Respiratory mucosa covered fibroadipose tissue exhibiting cartilage and minor salivary glands (a–c). The underlying soft tissue shows a neoplastic lesion with nested architecture. The lesional cells are arranged in organoid nests and interconnecting cords of cells exhibiting typical “Zellballen pattern”. The neoplastic cells are large, polygonal to round, having hyperchromatic nuclei showing occasional enlargement with salt and pepper chromatin and abundant eosinophilic granular cytoplasm. Scant mitoses are appreciated with intervening thin vasculature. The neoplastic cords and nests are outlined by delicate sustentacular cell network. (d) The tumor shows tongue-like protrusions into adjacent thyroid tissue.

**Figure 3:**
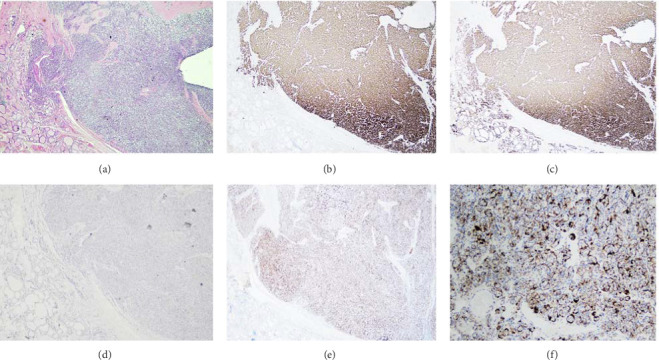
((a) H&E 10x) Tumor with adjacent thyroid parenchyma. (b) Immunohistochemical stain Synaptophysin is diffusely positive in the neoplastic cells. (c) Immunohistochemical stain CD56 is diffusely positive in the neoplastic cells. (d) Immunohistochemical stain Calcitonin is negative in the neoplastic cells. ((e, f) (20x)) Immunohistochemical stain S100 protein highlights the spindle sustentacular cells at the periphery of the cellular nests.

The final histopathology report showed a neoplastic lesion with nested architecture (Figure 2). The lesional cells were arranged in nests and interconnecting cords of cells exhibiting a typical zellballen pattern. The neoplastic cells were large, polygonal to round, and hyperchromatic nuclei showing occasional enlargement with salt and pepper chromatin and abundant eosinophilic granular cytoplasm. Scant mitoses were appreciated with intervening thin vasculature. The neoplastic cords and nests were outlined by delicate sustentacular cell network highlighted with immunohistochemical stain S100. Immunohistochemistry showed synaptophysin, CD56, GATA-3 positive, and S100 highlights supporting sustentacular cells (Figure 3). The mass was negative for TTF-1 and calcitonin favouring a diagnosis consistent with PGL extending into the right thyroid lobe.

The patient was reviewed by endocrinology and workup was negative for any secretory tumor. A computed tomography (CT) scan showed nonvisualization of the thyroid gland. No residual disease was appreciated. She is being followed regularly and is in stable condition.

## 3. Discussion

PGLs are tumors that originate from endocrine cells. They appear along the paraganglia route, migrating from the base of the skull to the pelvic floor during embryonic development [4].

Most head and neck PGLs (HNPGLs) originate from the carotid body, glossopharyngeal or vagus nerves, or the jugular bulb, with up to 60% of all PGLs in the head and neck being carotid body PGLs [5].

Hereditary HNPGLs account for 40% of all cases and typically manifest ten years earlier than sporadic cases. A large majority of HNPGLs are benign, with approximately 19% of them being malignant. Previous reports have shown metastases as a reliable indicator of malignancy [4].

HNPGLs normally present as a painless, slow-growing mass. Most are asymptomatic and found incidentally as nonfunctional thyroid nodules on imaging [3]. As their size increases, patients may present with complaints of dysphagia, hoarseness, and other cranial nerve deficits [4]. Thyroid PGLs, in particular, are rare tumors with a strong female predominance, typically presenting around age 48. A known risk factor for the development of PGLs is chronic hypoxia [5].

Establishing the diagnosis of PGLs traditionally requires biochemical evidence of catecholamine production by the tumor. Fractionated metanephrines such as normetanephrine have a higher diagnostic sensitivity compared to parent catecholamines when tested for in plasma or urine [6]. However, PGLs located in the thyroid are associated with the parasympathetic nervous system and are often missing an essential enzyme, tyrosine hydroxylase, which is used in dopamine synthesis. Therefore, these patients are often asymptomatic, as minimal to no catecholamines are released from the tumor [7]. Regardless, it is recommended that all patients with newly diagnosed PGLs should undergo a biochemical evaluation for catecholamine excess in order to prevent intraoperative complications such as catecholamine storm. Patients with thyroid PGLs tend to be euthyroid and serum antibodies for antithyroglobulin, antiperoxidase, and parathyroid hormone are usually negative [3].

Ultrasound with FNAC is normally sensitive and specific to thyroid lesions, yet due to the rarity of PGLs in this region, it is commonly misdiagnosed as a follicular or medullary lesion. Ultrasound with Doppler findings are generally nonspecific and describe PGLs as well-defined, hypoechoic, heterogenous, and hypervascular lesions [8]. Follicular lesions such as adenomas tend to lack internal flow on Doppler imaging and instead demonstrate encapsulation which presents as a “halo” on ultrasound. Currently, no study directly compares vascularity of follicular adenomas with PGLs yet adenomas are considered to lack the same kind of hypervascularity seen in PGLs. If morphology of a lesion is suspicious for a follicular lesion in TBSRTC with very high vascularity on Doppler ultrasound and lacks features consistent with adenoma, then PGL should be considered. Immunohistochemistry can further test and support this possibility [9].

Differentiating PGLs from MTC is slightly more definitive as MTC usually exhibits marked hyperechogenicities that resemble calcifications whereas paragangliomas lack any kind of calcified findings. Furthermore, metastasis to nearby lymph nodes is more characteristic of MTC that are greater than 1 cm in size compared to PGLs which are usually benign and do not metastasize. Similar to follicular lesions, there are no concrete or guaranteed ultrasound findings that can definitively distinguish PGLs from follicular or medullary neoplasms [9].

Fine needle aspiration of PGLs typically shows a predominance of blood, with cells appearing in various forms such as round, plasmacytoid, or spindled. These cells are arranged in clusters, acinar formations, isolated patterns, or syncytial groups, with nuclei that can be uniform or variable pleomorphic. Additionally, PGLs may display nuclear grooves and intranuclear pseudoinclusions [10].

Due to their rarity, the creation of a robust immunohistochemical profile is necessary to help diagnose thyroid PGLs. Normal calcitonin and carcinoembryonic antigen (CEA) levels help to rule out medullary carcinoma [11]. However, cases where PGLs produce elevated calcitonin and CEA do exist. In these scenarios, biomarkers such as synaptophysin, chromogranin A, neuron specific enolase, CD 56 and S-100 can help differentiate PGL from MTC [12]. Parathormone, EMA, TTF1, and AE1/AE3 are usually negative [8].

PGLs are hypervascular tumors that show significant enhancement on both CT and MRI scans. On CT, they exhibit pronounced enhancement of intratumoral vessels after contrast administration. On MRI, PGLs are typically low in signal on T1-weighted images and intermediate-to-high in signal on T2-weighted images, with intense gadolinium enhancement. A distinctive feature of these tumors is the flow signal voids, which create a characteristic salt-and-pepper appearance on spin-echo sequences [13]. To diagnose PGLs, positron emission tomography/CT (PET/CT) is the preferred imaging method. The latest Endocrine Society Guidelines recommend using 18F-fluoro hydroxyphenylalanine PET/CT as the optimal functional imaging approach [14].

Surgical excision with either a partial or total thyroidectomy, depending on the degree of neoplastic malignancy, is the preferred course of treatment. The existence of metastases to lymph nodes or other organs is considered while evaluating this trait. Scoring systems for predicting metastases have been created but are limited to PGLs of the adrenals and do not extend to the head and neck region [5]. Preoperative evaluation of airway involvement is critical in head and neck malignancies to guide surgical planning and minimize morbidity. Imaging modalities such as contrast-enhanced CT and procedures like bronchoscopy can accurately assess tracheal invasion preoperatively, potentially preventing unnecessary airway resections and associated complications such as subglottic stenosis. Incorporating such assessments may enhance decision-making in complex airway management.

An adjuvant radiation treatment on the laterocervical lymph nodes may be recommended when an aggressive behavior is observed. Given its unexpected behavior and continuous recurrence that has been recorded between 5 and 15 years after first surgery, patients diagnosed with thyroid PGLs should have regular and ongoing clinical and imaging follow-ups [11]. This includes biochemical screening on a yearly basis along with a full body MRI every two to 3 years [5].

## Data Availability

All data generated during this study is included in this article.
